# Cigarette toxicity triggers Leber's hereditary optic neuropathy by affecting mtDNA copy number, oxidative phosphorylation and ROS detoxification pathways

**DOI:** 10.1038/cddis.2015.364

**Published:** 2015-12-17

**Authors:** L Giordano, S Deceglie, P d'Adamo, M L Valentino, C La Morgia, F Fracasso, M Roberti, M Cappellari, G Petrosillo, S Ciaravolo, D Parente, C Giordano, A Maresca, L Iommarini, V Del Dotto, A M Ghelli, S R Salomao, A Berezovsky, R Belfort, A A Sadun, V Carelli, P Loguercio Polosa, P Cantatore

**Affiliations:** 1 Department of Biosciences, Biotechnologies and Biopharmaceutics, University of Bari, Bari, Italy; 2 Department of Reproductive Sciences, Medical Genetics, Development and Public Health, University of Trieste, Trieste, Italy; 3 IRCCS-Burlo Garofolo Children Hospital, Trieste, Italy; 4 IRCCS Institute of Neurological Sciences of Bologna, Bellaria Hospital, Bologna, Italy; 5 Department of Biomedical and NeuroMotor Sciences (DIBINEM), Neurology Unit, University of Bologna, Bologna, Italy; 6 Institute of Biomembranes and Bioenergetics (IBBE) National Research Council (CNR), Bari, Italy; 7 Vectis s.r.l. Cava dei Tirreni (Salerno), Italy; 8 Department of Radiological, Oncological and Pathological Sciences, Sapienza University of Rome, Rome, Italy; 9 Department of Pharmacy and Biotechnology, University of Bologna, Bologna, Italy; 10 Department of Ophthalmology, and Visual Sciences, Paulista School of Medicine Federal University of Sao Paulo—UNIFESP, Sao Paulo, Brazil; 11 Doheny Eye Institute, University of California Los Angeles (UCLA), Los Angeles, CA, USA

## Abstract

Leber's hereditary optic neuropathy (LHON), the most frequent mitochondrial disease, is associated with mitochondrial DNA (mtDNA) point mutations affecting Complex I subunits, usually homoplasmic. This blinding disorder is characterized by incomplete penetrance, possibly related to several genetic modifying factors. We recently reported that increased mitochondrial biogenesis in unaffected mutation carriers is a compensatory mechanism, which reduces penetrance. Also, environmental factors such as cigarette smoking have been implicated as disease triggers. To investigate this issue further, we first assessed the relationship between cigarette smoke and mtDNA copy number in blood cells from large cohorts of LHON families, finding that smoking was significantly associated with the lowest mtDNA content in affected individuals. To unwrap the mechanism of tobacco toxicity in LHON, we exposed fibroblasts from affected individuals, unaffected mutation carriers and controls to cigarette smoke condensate (CSC). CSC decreased mtDNA copy number in all cells; moreover, it caused significant reduction of ATP level only in mutated cells including carriers. This implies that the bioenergetic compensation in carriers is hampered by exposure to smoke derivatives. We also observed that in untreated cells the level of carbonylated proteins was highest in affected individuals, whereas the level of several detoxifying enzymes was highest in carriers. Thus, carriers are particularly successful in reactive oxygen species (ROS) scavenging capacity. After CSC exposure, the amount of detoxifying enzymes increased in all cells, but carbonylated proteins increased only in LHON mutant cells, mostly from affected individuals. All considered, it appears that exposure to smoke derivatives has a more deleterious effect in affected individuals, whereas carriers are the most efficient in mitigating ROS rather than recovering bioenergetics. Therefore, the identification of genetic modifiers that modulate LHON penetrance must take into account also the exposure to environmental triggers such as tobacco smoke.

Leber's hereditary optic neuropathy (LHON) is among the most frequent mitochondrial diseases, affecting about 1 in 35 000–60 000 in Europe.^1, 2^ LHON is associated in over 90% of cases with one of three common mitochondrial DNA (mtDNA) point mutations affecting the Complex I subunit genes ND4 (m.11778G>A), ND1 (m.3460G>A) and ND6 (m.14484 T>C), usually occurring in homoplasmic fashion^3, 4^ (100% of mtDNA is mutant). This maternally inherited blinding disorder is caused by selective degeneration of retinal ganglion cells, particularly those originating the small axons composing the papillomacular bundle, which leads to optic atrophy.^5, 6, 7^ Clinically, a subacute loss of central vision develops in weeks/months, mostly affecting young adult men, with a peculiar pattern of fiber depletion^8^ and a relatively predictable natural history of visual function decline.^9^ Exceptions apply to age of onset, with childhood or late cases,^10, 11^ to propensity in recovering vision, more frequent with the m.14484 T>C mutation,^12^ and to clinical expression that in a subset of patients may be more widespread.^4^

The mtDNA mutations are necessary but not sufficient to cause LHON,^13^ with penetrance being on average about 50% for males and 10% for females. The association of specific mtDNA haplotypes of haplogroup J with the m.14484 T>C and m.11778G>A mutations has been consistently documented in patients of European descent, indicating that mtDNA background modulates to a certain extent disease penetrance.^14, 15^ However, in a prototypical LHON maternal lineage, despite all the individuals carry the homoplasmic mtDNA mutation regardless the haplogroup, only some develop the disease, pointing to further factors that may be genetic and environmental.^16^ Thus, male prevalence and incomplete penetrance remain the two investigated and problematic issues in LHON. Both issues have been recently mechanistically related to the efficiency of compensatory mitochondrial biogenesis.^17, 18^ Estrogens ameliorate mitochondrial dysfunction by activating mitochondrial biogenesis, suggesting that females are naturally protected during their fertile period.^17, 19^ Furthermore, by studying different experimental systems (blood cells, skeletal muscle, skin-derived fibroblasts and ocular tissue) we found that the unaffected mutation carriers had a significantly higher mtDNA copy number and mitochondrial mass compared with their affected relatives,^18^ indicating that efficiently increasing mitochondrial biogenesis may overcome the pathogenic effect of the mtDNA mutation. Recently, others obtained similar results in different LHON cohorts.^20^ Notwithstanding, nuclear modifiers remain elusive. In particular, association of LHON with genetic variants was not consistent across different studies.^18, 21^ Similarly inconsistent was the association with chromosome X-linked loci, hypothesized to explain male prevalence.^22, 23, 24^

Several other factors have been implicated in LHON, including exposure to cigarette smoke, alcohol and chemical toxins, head trauma, acute physical illness, psychological stress, antiretroviral and antituberculosis drugs.^4, 25^ These and other environmental factors can have a triggering role in LHON pathogenesis. For example, *in vitro* exposure to 2,5 exanedione had a toxic effect on LHON cybrid cells, with an increased sensitivity if they harbored a haplogroup J background.^26^ A major environmental trigger of LHON is cigarette smoke; Sadun *et al.*^27^ and Kirkman *et al.*^25^ showed that LHON penetrance is significantly increased in smokers, independently of gender and alcohol intake.

In the current study, we explored further the effect of cigarette smoking in LHON, showing in white blood cells from patients of large LHON cohorts, and in skin-derived fibroblasts, that cigarette derivatives exert their toxicity by depressing mtDNA copy number and oxidative phosphorylation (OXPHOS). However, unaffected mutation carriers displayed the most efficient capacity for reactive oxygen species (ROS) detoxification, which was not hampered by exposure to cigarette derivatives.

## Results

### Tobacco smoking significantly depresses mtDNA copy number in LHON patients

**Figure 1:**
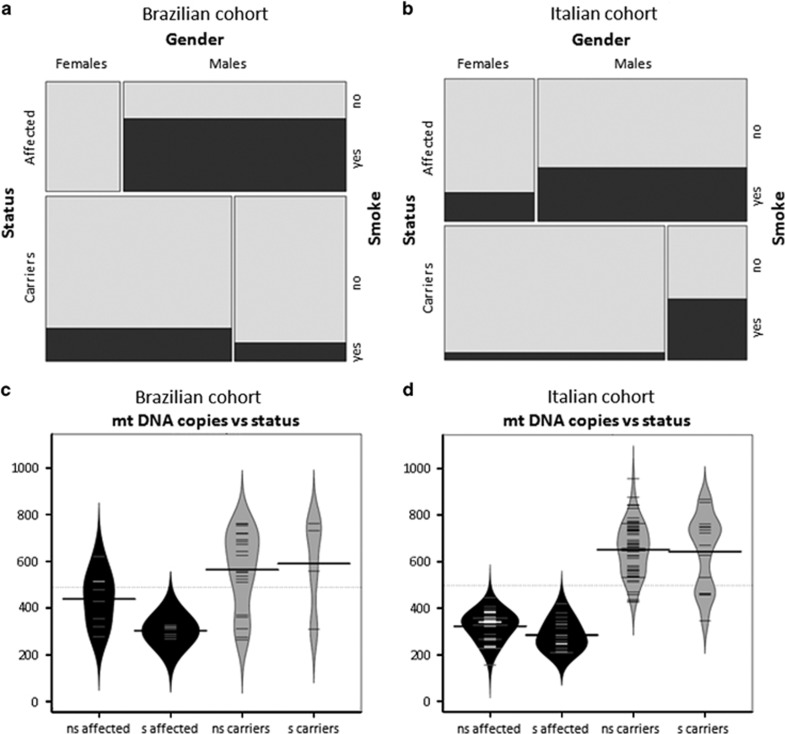
Mosaicplots and beanplots of the Brazilian SOA-BR family and Italian cohort of LHON families. (**a** and **b**) Mosaicplots of the relation between ‘Gender', ‘Status' and ‘Smoke' in the Brazilian SOA-BR family and in the Italian cohort, respectively. Smokers are indicated with black rectangles and non-smokers in light gray. The areas of the boxes in the plot are proportional to the cell frequencies of the contingency table for the three variables (**c** and **d**) Beanplots representing the distribution of mtDNA copies between smokers (s) and non-smokers (ns) in the Brazilian SOA-BR family and in the Italian cohort, respectively. The short lines represent individual data points and the longest lines represent the mean of the values

We previously studied the large SOA-BR LHON family from Brazil and an Italian cohort of 39 LHON families demonstrating that a significant difference characterizes unaffected mutation carriers compared with affected individuals in terms of mtDNA copy number and mitochondrial biogenesis, both of which are activated more efficiently in unaffected mutation carriers.^18^ Having now available mtDNA copy number and cigarette smoke habits on the same subjects, we stratified the mtDNA quantitative data by smokers/non-smokers in affected and unaffected mutation carriers, considering separately the SOA-BR family and the Italian cohort. A descriptive analysis reveals some differences between the two cohorts. For example, all the affected women in the SOA-BR cohort were non-smokers (Figure 1a), whereas 20% of the affected women in the Italian cohort were smokers (Figure 1b). Thus, to take into account the composition of the two cohorts and their different genetic background, we conducted a random-effects meta-regression, where both within-study and between-study variations are considered.

In the first analysis, we estimated the differences in the distribution of smokers between affected individuals and unaffected mutation carriers. In both cohorts there was an increased percentage of smokers in the affected group (Figure 1a and b) and the association result from the meta-analysis was highly significant (*P*=0.009; Supplementary Figure1A). Subsequently, we analyzed the mtDNA copy number in the carrier and affected groups, considering separately smokers and non-smokers. As shown in Figure 1c and d, the affected smokers had a lower mtDNA copy number as compared with affected non-smokers and with the unaffected mutation carriers, with a similar tendency in both the Brazilian family (Figure 1c) and the Italian cohort (Figure 1d). The unaffected mutation carriers smokers displayed similar values as the non-smokers. Pooling all the data for the affected individuals in a meta-analysis, the reduction of mtDNA copy number in affected smokers was significant (*P*=0.026; Supplementary Figure 1B).

Taken together, these results show that cigarette smoking has a direct impact on LHON penetrance and that it may affect the mtDNA copy number and the compensatory activation of mitochondrial biogenesis, sparing the unaffected carriers.

### Cigarette smoke condensate affects mtDNA copy number of fibroblasts

To directly test the effects of cigarette smoking on LHON, we used fibroblasts derived from skin biopsies of controls, LHON affected individuals and unaffected mutation carriers. Cells were incubated with cigarette smoke condensate (CSC), an extract that contains many toxic compounds including polycyclic aromatic hydrocarbons such as benzo-a-pyrene, phenolic compounds, tobacco-specific nitrosoamine, aromatic amines, quinones and heavy metals.^28^ We first analyzed fibroblasts viability after exposure for 72 h to increasing amounts of CSC, finding that cell viability, measured by the SRB cytotoxic assay, remained unchanged up to a CSC concentration of 120 *μ*g/ml, and then declined in all cell categories (Supplementary Figure 2). Therefore, in the subsequent experiments we chose to treat cells for 72 h with amounts of CSC within 120 *μ*g/ml, avoiding higher concentrations for which CSC toxicity would affect cell viability masking the potentially different response to cigarette smoke of the three groups of cells.

**Figure 2:**
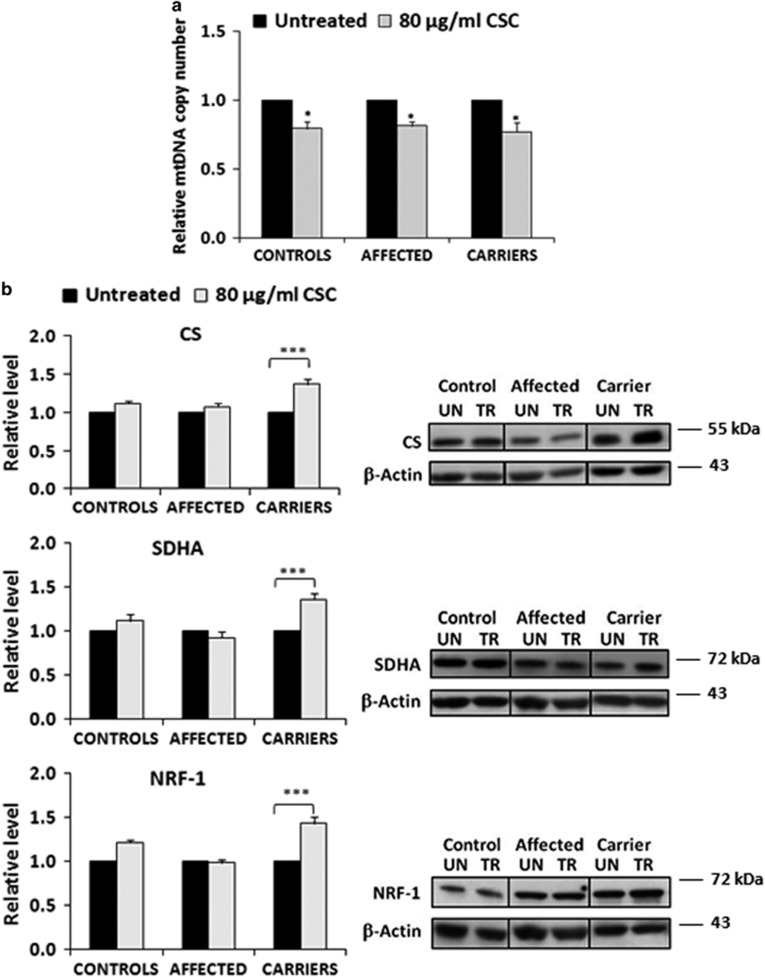
Effect of CSC on the fibroblasts mtDNA copy number and on the content of marker proteins of mitochondrial mass and biogenesis. Five samples for each cell type were used and results are the average of quadruplicate determination for each sample. The histograms report the mean value (±S.E.M.) normalized to the untreated samples. *P (treated *versus* untreated) <0.05. ***P (treated *versus* untreated) <0.001. (**a**) Effect of CSC on mtDNA copy number. Exposure to CSC decreases the level of mtDNA in all three cell types (**b**) Effect of CSC on the content of CS, SDHA and NRF-1. CSC increases significantly the level of the three proteins only in carriers. No substantial change is observed in affected and controls. A representative western blotting is also shown (UN, untreated; TR, treated)

To evaluate the effect of CSC on mtDNA copy number, we extracted total DNA from cells incubated with 80 *μ*g/ml of CSC and measured the ratio between mtDNA and nuclear DNA by qPCR. Figure 2a shows that exposure to CSC decreased the mtDNA copy number by about 25–30% in all three groups of cells. This result confirms *in vitro* that tobacco toxicity induces mtDNA copy number reduction.

### Unaffected mutation carriers display the most efficient compensatory activation of mitochondrial biogenesis after exposure to CSC

Having established that exposure to CSC leads to reduction of mtDNA copy number in fibroblasts, we next investigated how CSC impacts on the level of proteins marking mitochondrial mass and biogenesis such as citrate synthase (CS), the subunit A of succinate dehydrogenase (SDHA) and nuclear respiratory factor-1 (NRF-1). Figure 2b shows that exposure to 80 *μ*g/ml of CSC caused a significant increase in the level of all three proteins in fibroblasts from unaffected mutation carriers, whereas there was no significant variation in fibroblasts from controls and affected individuals. We also measured the CS activity and found that it exhibited the same trend of the protein content (data not shown).

These results indicate that, despite the observed reduction in mtDNA copy number, unaffected mutation carriers challenged by CSC can still orchestrate efficiently a compensatory activation of mitochondrial biogenesis.

### CSC exposure affects fibroblast bioenergetics

**Figure 3:**
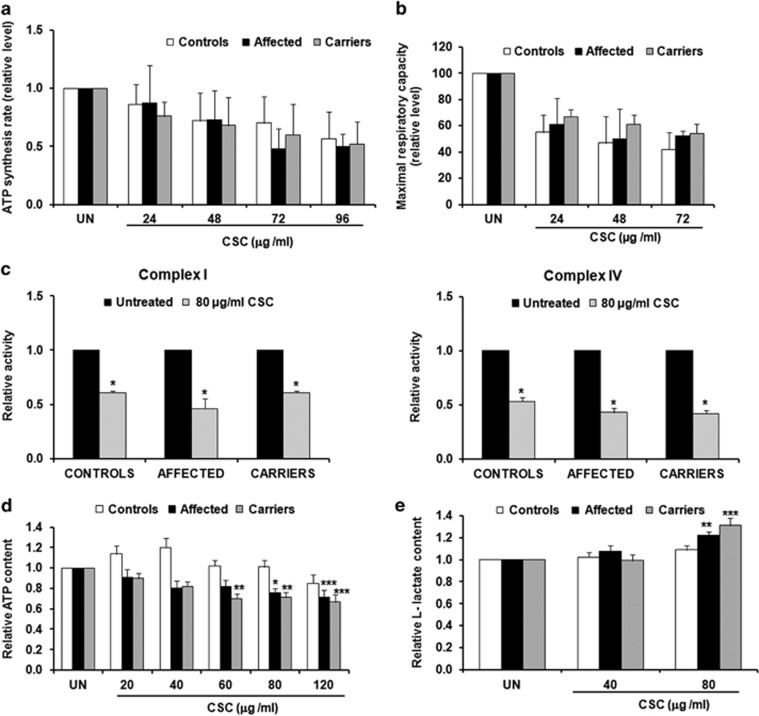
Effect of CSC on fibroblasts bioenergetics. (**a**) Rate of Complex I-dependent ATP synthesis. The values (mean±S.E.M.) are normalized to the respective untreated samples (UN). Although not statistically significant, the data (average of measures done in three samples for each cell type) show that exposure to CSC decreases the rate of ATP synthesis in all three cell types (**b**) Oxygen consumption rate (OCR) in adherent cells. The values, average percentage (±S.E.M.) of residual maximal respiration capacity with respect to untreated cells (UN) show that CSC decreases the OCR in all three cell types with controls showing a slightly lower decrease. Each value was the mean based on at least two independent determinations obtained from two different cell lines (**c**) Activity of respiratory Complex I and IV. Data are the average value (±S.E.M.) of three determinations on four different samples for each cell type. The histogram shows the ratio of the complex activities between treated and untreated samples. *P (treated *versus* untreated) <0.05. CSC treatment causes a decrease of the activities of both complexes. The decrease is slightly higher in the affected subjects (**d**) Intracellular level of ATP. The values (average±S.E.M. of four measures done in five samples for each cell type) are normalized to the respective untreated samples (UN). *P (treated *versus* untreated) <0.05; **P<0.01. Increasing amounts of CSC cause a significant decrease of ATP content in affected and carriers. No significant variation is observed for the control lines (**e**) l-lactate level. The values (average±S.E.M. of four measures done in five samples for each cell type) are normalized to the respective untreated samples (UN). *P (treated *versus* untreated) <0.05; **P<0.01. l-lactate increases significantly in affected and carriers at 80 *μ*g/ml of CSC. Controls do not show a significant increase although they exhibit an increasing trend

To study the effect of CSC on bioenergetics, we assessed the rate of ATP synthesis driven by Complex I substrates and oxygen consumption of fibroblasts immediately after the addition of incremental amounts of CSC. These treatments progressively decreased ATP synthesis rate down to about 50% with a slight prevalence in fibroblasts from LHON affected individuals (Figure 3a). Similarly, the maximal respiration capacity was decreased in all cell lines (Figure 3b), demonstrating that CSC impacts on OXPHOS.

We next measured the activities of respiratory Complexes I and IV in mitochondria isolated from fibroblasts treated for 72 h with 80 *μ*g/ml of CSC. Figure 3c shows that Complex I activity was markedly reduced in all three groups. Fibroblasts from LHON affected subjects exhibited the strongest reduction (by about 55%), whereas fibroblasts from unaffected mutation carrier and controls were reduced by about 40%, with no statistical differences among groups. Similarly, Complex IV activity was reduced in carriers and affected by 57%, and slightly less in controls by 47%. To evaluate the overall impact of the OXPHOS impairment, we measured the total ATP content in fibroblasts grown for 72 h in the presence of incremental CSC amounts (from 20 to 120 *μ*g/ml). ATP content was not significantly impacted by CSC exposure in controls (Figure 3d), whereas in both affected and unaffected mutation carriers CSC decreased the ATP level by about 35%. Finally, we measured the level of l-lactate excreted in the medium. We found that the lactate level was slightly increased in both affected and unaffected mutation carriers (Figure 3e). This suggests that LHON mutant cells, notwithstanding pushing for a greater energy metabolism by means of the glycolytic flux, failed to reconstitute the ATP content.

Altogether these results clearly indicate that CSC affects fibroblast bioenergetics, impacting mostly on the LHON mutant cells.

### CSC and ROS damage

**Figure 4:**
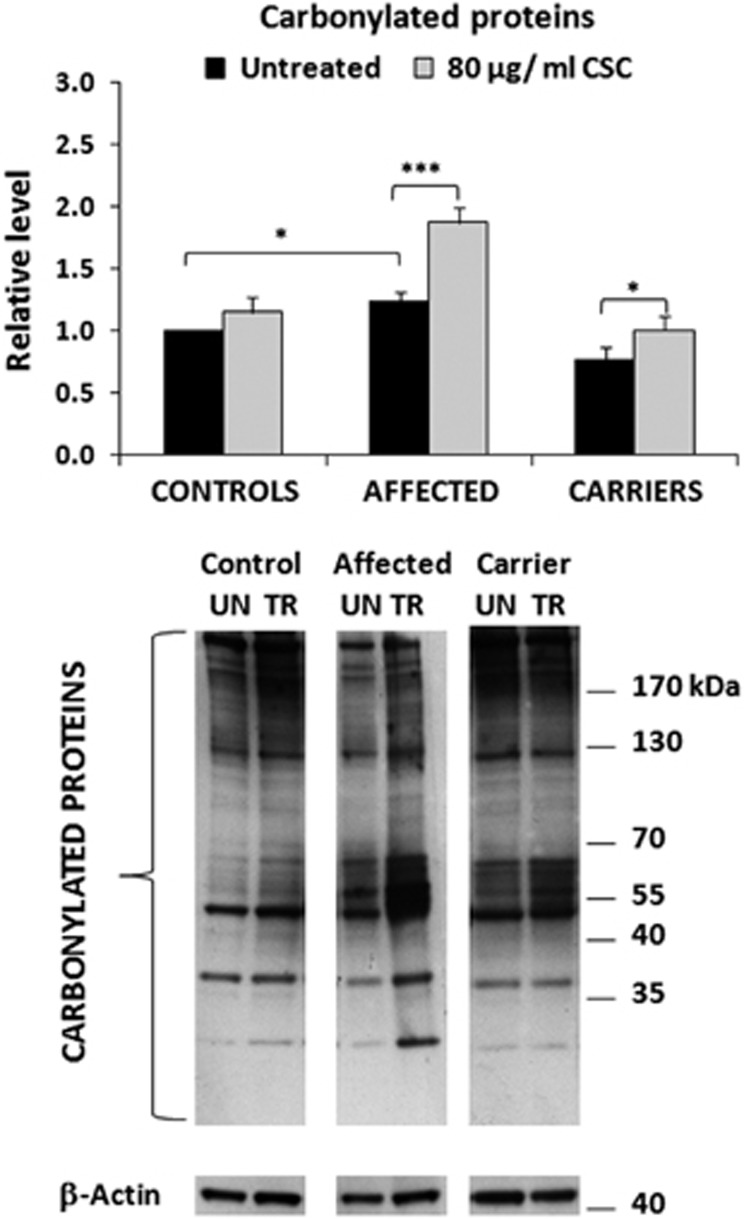
Effect of CSC on the level of carbonylated proteins. Five samples for each cell type were used and experiments were performed in quadruplicate. All values (average±S.E.M.) are normalized to the untreated control sample. A representative western blotting is also shown (UN, untreated; TR, treated). *P<0.05; ***P<0.005. The histogram shows that in basal conditions the relative level of carbonylated proteins is higher in the affected cells compared with the other cell types. CSC treatment induces an increase of carbonylated proteins especially in affected

Previous studies have demonstrated that LHON cybrids have an increased ROS production compared with controls.^17, 29^ Moreover, LHON cybrids have a reduced antioxidant defense that is highlighted by the exposure to stressful condition such as growth in galactose medium.^30^ Carbonyl groups of the proteins are a major product of a variety of ROS-mediated oxidation reactions, so that their measurement provides a generalized and integrated evaluation of the oxidative damage. As CSC is a source of exogenous ROS, we first evaluated the effects of ROS production in untreated fibroblasts from the three groups (basal conditions) by measuring the amount of carbonylated proteins. Figure 4 shows that carbonylated proteins are more abundant in LHON affected fibroblasts than in controls, thus confirming the previously observed increase of ROS production in LHON cybrids.^17, 29, 30^ Interestingly, fibroblasts from unaffected mutation carriers had the lowest content of carbonylated proteins compared with both LHON affected and also controls. This suggests that unaffected mutation carriers display a more efficient antioxidant defense compared with LHON affected individuals. Then, CSC treatment increased the level of carbonylated proteins, particularly in fibroblasts from LHON affected individuals (by about 50%), whereas unaffected mutation carriers showed only a moderate increase, and controls did not change significantly (Figure 4). Considering that fibroblasts from LHON affected individuals already had a higher ROS damage compared with unaffected mutation carriers, the CSC treatment further enhanced this difference.

### CSC and ROS detoxification

**Figure 5:**
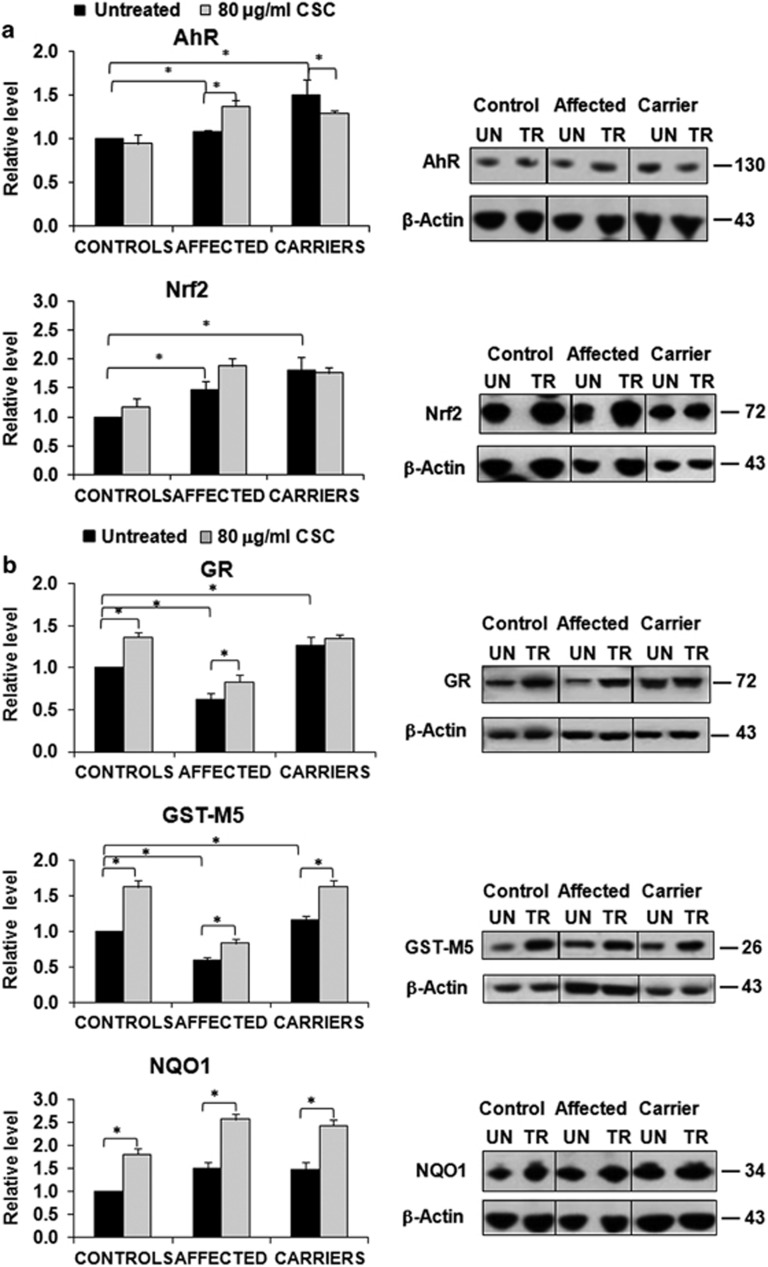

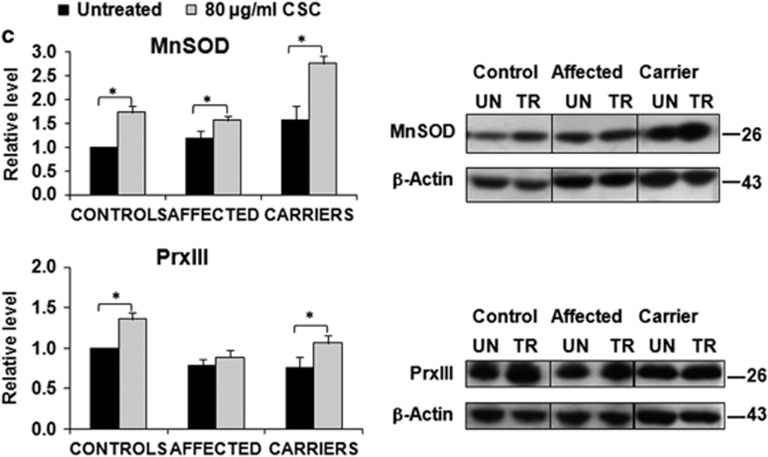
Effect of CSC on the level of proteins involved in ROS detoxification. Five samples for each cell type were used and experiments were performed in quadruplicate. All values (average±S.E.M.) are normalized to the untreated control sample. A representative western blotting is also shown. *P<0.05. (**a**) Content of AhR and Nfr2. The histogram shows that in the untreated samples both proteins have a higher level in carriers. CSC treatment induces a slight increase especially in the affected (**b**) Content of NQO1, GR and GST-M5. The histogram shows that in the basal conditions carriers exhibit a higher level of the three proteins. CSC treatment causes a slight increase of the proteins in the three cell types (**c**) Content of MnSOD and Prx III. In the untreated samples MnSOD is higher in carriers, while Prx III does not show significant variations among the three cell types. CSC treatment increases the level of the two proteins especially in carriers and controls

To investigate how variable levels of ROS damage relates to the efficiency of xenobiotic detoxification pathways, we first assessed two nuclear transcription factors involved in these processes, that is, the aryl hydrocarbon receptor (AhR) and the Nuclear factor erythroid 2-related factor 2 (Nrf2). AhR is a member of the basic helix-loop-helix Per-Arnt-Sim transcription family and its expression is essential to regulate cell proliferation and to prevent mitochondrial dysfunction caused by cigarette smoke.^31^ In particular, it has been shown that AhR regulates the expression of manganese superoxide dismutase (MnSOD). Nrf2 is a transcription factor that is activated by oxidative stress and binds to the antioxidant response elements of target genes, including those involved in glutathione synthesis, elimination of ROS, detoxification of xenobiotics and drug transport.^32^ We measured the level of AhR and Nrf2 in basal conditions and found that both factors were higher in unaffected mutation carriers than in LHON affected and controls (Figure 5a). This result highlights that antioxidant efficiency is greater in unaffected mutation carriers. CSC treatment (80 *μ*g/ml) only slightly modified this pattern increasing the AhR level in the fibroblasts from affected individuals. This indicates that in the presence of smoke derivatives LHON mutant cells express the genes regulating xenobiotic detoxification at a higher level compared with controls.

We next measured the level of three cytosolic proteins involved in ROS detoxification, such as NADPH dehydrogenase quinone 1 (NQO1), glutathione S transferase mu5 (GST-M5) and glutathione reductase (GR). In basal conditions the level of these proteins was slightly higher in unaffected mutation carriers, compared with controls and LHON affected. CSC treatment increased their levels in all cell types at a similar extent (Figure 5b).

Finally, we evaluated the antioxidant response within mitochondria, assessing the levels of MnSOD and peroxiredoxin III (Prx III). In basal conditions (Figure 5c) MnSOD was highest in unaffected mutation carriers, reflecting the AhR level. Prx III did not display significant variations among the three cell types, being slightly lower in unaffected mutation carriers and LHON affected compared with controls. CSC treatment induced an increase of both proteins in all cell types, with unaffected mutation carriers and controls exhibiting the highest levels. The overall outcome of these studies is that unaffected mutation carriers, previously shown to exhibit a compensatory increase of mitochondrial biogenesis,^18^ also display a more efficient system for ROS detoxification compared with both LHON affected and control individuals.

## Discussion

Previous epidemiological studies from our and other laboratories^25, 27^ suggested that cigarette smoke is significantly associated with LHON penetrance. However, although several reports indicated that cigarette smoke has various deleterious effects on mitochondrial function^33, 34, 35, 36^ its impact on subjects carrying the LHON mutations has never been previously investigated. Here, for the first time, we report results that link directly the effects of cigarette smoke with LHON penetrance. First, by crossing our pre-existing results on mtDNA copy number with the information on cigarette smoke exposure of the same subjects from two separate LHON cohorts,^18^ we documented not only that tobacco smoke was significantly associated with LHON affected individuals, but also that among the affected individuals, smokers had significantly reduced mtDNA copy number. Thus, tobacco smoke further reduced the already limited efficiency of LHON affected individuals to cope with the impaired respiratory function. Then we studied the effect of cigarette extracts on mtDNA copy number and other indexes of mitochondrial biogenesis, bioenergetics and oxidative stress in fibroblasts obtained from controls, LHON affected patients and unaffected mutation carriers. We found that tobacco toxicity not only reduces mtDNA copy number in all cell types, but also directly affects mitochondrial function especially in mutant cells. It remains unclear through which mechanism and signaling pathway tobacco toxicity exerts its depressing effect on mtDNA copy number. We consider this effect as additive to the more direct and specific inhibition of single respiratory complexes (I and IV), as we documented. Tobacco smoke-induced reduction of respiratory complex activities has already been shown in various systems, likely due to specific CSC constituents.^37, 38, 39^ Unaffected mutation carriers were better able to deal with tobacco toxicity by most efficiently activating mitochondrial biogenesis. However, this was not sufficient to compensate for the overall decline of mitochondrial bioenergetics, as CSC exposure caused a decrease of the ATP content in LHON mutant cells, including unaffected mutation carriers, whereas in basal conditions carriers and controls had a similar ATP content, higher than LHON affected.^18^

In spite of the diminished content of ATP specific to LHON mutant cells, we found that respiratory enzyme activities and oxygen consumption rate were decreased in all cell types. This may be due to a combination of different effects. The unchanged ATP level in controls may be ascribed to a ‘threshold effect'. It is well-known that decrease in activity of the respiratory complexes reflects on OXPHOS efficiency only when it exceeds a certain threshold,^40, 41^ which may vary according to the experimental conditions and is usually high (over 80% impairment in OXPHOS). Conversely, the decrease of ATP content in the unaffected mutation carriers despite their compensatory skills may be explained considering that carriers, by exhibiting the highest ROS detoxifying activity among the three groups of cells (see below), consume the most ATP in xenobiotic solfatation and methylation reactions, as well as in the synthesis of gluthatione.^42, 43^ Furthermore, the conversion of oxidized to reduced glutathione requires NADPH, the reduced coenzyme is mainly provided by the mitochondrial transhydrogenase activity that, is energy dependent and driven by the electrochemical proton gradient.^44^

It has been shown in cybrids that LHON mutations increase ROS production.^17, 29, 30^ However, the use of cybrids, in which a uniform nuclear background derives from the original osteosarcoma cell line,^45^ does not allow distinguishing between unaffected mutation carriers and LHON affected. We overcome this limit by using cultured fibroblasts, which retain the different nuclear background. Our results show that under basal conditions the levels of protein carbonylation in fibroblasts from unaffected mutation carriers was significantly lower compared with LHON affected and controls. Concordantly, the level of several antioxidant enzymes was higher in carriers compared with the other two cell types. Therefore, carriers not only activate mitochondrial biogenesis better than LHON affected individuals, but display also a better capability to activate the antioxidant machinery. Even in the presence of CSC, carriers exhibited the best compensatory response to the increased oxidative stress compared with cells from LHON affected individuals, displaying less carbonylated proteins and higher level of several detoxifying enzymes.

The increased mitochondrial biogenesis and efficient ROS protection displayed by carriers may depend on interlinked pathways. Interestingly, several reports highlighted a relationship between the Nrf2–NQO1 pathway and mitochondrial biogenesis. Kwon *et al.*^46^ reported that loss of both proteins decreased membrane potential and altered mitochondrial integrity, whereas Piantadosi *et al.*^47^ showed that among the genes activated by Nrf2 there is also NRF-1, which has a key role as master regulator of mitochondrial biogenesis and is increased in carriers treated with CSC. Therefore, Nrf2 activation could be a part of the signaling pathway leading to efficient mitochondrial biogenesis. NQO1 is responsible for using NAD(P)H as an electron donor to catalyze the 2-electron reduction of damaging substances and quinones. The formation of NAD^+^ has been recently shown to fuel mitochondrial biogenesis through Sirt1 and activation of PGC1*α*.^48, 49, 50^ Therefore, the higher levels of proteins marking mitochondrial mass and mitochondrial biogenesis (NRF-1 and SDHA), exhibited by carriers in the presence of CSC, possibly depend on a high efficiency of the Nrf2–NQO1 axis in these cells.

The efficient activation of mitochondrial biogenesis and ROS protection pathways shown by LHON unaffected carriers is most probably due to differences in the nuclear genetic background distinguishing them from the LHON affected individuals. The genetic background, characterized by still unknown variants, also interacts efficiently with environmental challenges, such as exposure to tobacco toxicity. We recently found evidence^51^ that, among individuals carrying the LHON mutations, some are genetically primed to loose vision as young-adults in the absence of any environmental exposure, whereas others become affected with LHON only later, in mid-life, mostly because of prolonged (decades) heavy exposure to environmental triggers such as tobacco smoking. In this framework, the here observed decline of ATP levels in carrier fibroblasts exposed to CSC may be one of the effects of cigarette toxicity prompting conversion from unaffected mutation carrier to LHON affected. Thus, we believe that the genetic background priming some individuals to be affected early in life differs from the genetic background of individuals that become affected only after prolonged exposure to heavy tobacco smoking and from the genetic background that superprotects unaffected carriers who never become affected, despite the exposure to tobacco smoke. As a consequence, hunting for nuclear genetic modifiers must take into consideration not only covariates such as age and sex, but most importantly, environmental exposure to disease triggers. The results of association studies mixing LHON affected individuals genetically primed to the disease by nuclear genetic modifiers, with others affected only after an overwhelming long exposure to cigarette smoke, are profoundly biased by these confounding factors, diluting any meaningful genetic signal. This is possibly the reason for the prolonged failure to locate the LHON nuclear genetic modifiers.

In conclusion, the here reported results have a twofold relevance for understanding LHON pathogenesis. First, the major environmental trigger of LHON, tobacco smoking, directly affects the compensatory mechanism counteracting the pathogenic effects of LHON mutations, that is, the homeostatic regulation of mitochondrial biogenesis and maintenance of mtDNA copy number.^18^ The second important inference from our study is that the effect of tobacco toxicity clearly highlights how affected individuals are the less efficient in compensating for the respiratory defect induced by the LHON mutation, as opposed to unaffected mutation carriers, who are the most efficient. These results integrate with the previous studies by Giordano *et al.*,^17^ linking female estrogen protection with compensatory activation of mitochondrial biogenesis, which turns out to be a driving force for penetrance in both genders.^18^ Overall, this study poses the basis to correctly designing future investigations in the pursuit of nuclear genetic modifiers implicated in LHON penetrance.

## Materials and Methods

### LHON Brazilian pedigree and Italian cohort of LHON families

Blood samples and fibroblast cell lines were collected after informed consent according to the Declaration of Helsinki Principles, and approval of the Ethics Committee of the St. Orsola-Malpighi Polyclinic (University of Bologna) for the Italian cohort of LHON families, and the Sao Paulo Hospital (Federal University of Sao Paulo) for the Brazilian family SOA-BR. Both the Italian cohort and the SOA-BR family are the same reported in ref 18, and information about environmental triggers, including tobacco and alcohol consumption and toxic exposures, was obtained by telephone interviews and structured questionnaires, following the same criteria as described previously.^25^ Overall, we here investigated 25 affected and 38 unaffected mutation carriers from the maternal line of the SOA-BR pedigree, and 64 affected and 68 carriers from 39 unrelated Italian LHON pedigrees harboring one of the three common LHON mutations.^18^

### CSC preparation

The CSC is a fraction that derives from the solid part of the tobacco smoke and does not contain volatile components such as carbon monoxide. It was prepared through a smoking machine CERULEAN SM 450, using Coresta Monitor Test Pieces CM7 (Imperial Tobacco PLC, Hamburg, Germany). The particulate matter was collected on a glass fiber filter and its mass was determined by weighing the filter pad before and after the filtration. The particulate matter was then dissolved in dimethylsulfoxide at a final concentration of 17.4 mg/ml. CSC was stored at −20 °C until used.

### Fibroblasts cell lines and culture conditions

Fibroblast cell lines were established from skin biopsies taken from five controls, seven affected (six m.11778G>A and one m.14484 T>C) and six unaffected mutation carriers (m.11778G>A). Cells were grown in DMEM medium (EuroClone, Milano, Italy) containing 25 mM glucose and supplemented with 10% fetal bovine serum, 2 mM l-glutamine, 100 U/ml penicillin and 100 *μ*g/ml streptomycin, maintained at 37 °C in a humidified atmosphere with 5% CO_2_. The functional experiments were carried out on sub-confluent cell cultures obtained from a comparable number of culture passages (10–20). CSC was added at the concentrations indicated in the Figures and cells were kept for 72 h in the medium at 37 °C.

Detailed experimental procedures with cells (mitochondrial bioenergetics, SDS-PAGE, western blotting and quantification of carbonylated proteins) are available in the Supplementary Information.

### Extraction of total DNA and evaluation of mtDNA in blood cells and fibroblasts

Total DNA was extracted from an enriched white blood cell fraction. Quantitative Real-Time PCR (qRT-PCR) was used to assess mtDNA content in blood cells as reported in ref. 18.

Total fibroblast DNA was extracted using the Wizard Genomic DNA Purification Kit (Promega, Madison Wisconsin, USA) following the manufacturer's instructions. mtDNA copy-number quantification in fibroblasts was performed by a qRT-PCR method based on TaqMan probe chemistry (Applied Biosystems, Life Technologies, Monza, Italy), amplifying *MT-ND1* and *ACTB* genes. Primer sequences and PCR conditions are available upon request. The relative quantification of mtDNA was performed according the Pfaffl mathematical model.^52^

### Statistical analysis

Data were treated by analysis of variance with Tukey and Tukey's tests using SPSS Base 11.5 software (SPSS Inc., Chicago, IL, USA) to assess the significance of the differences observed between groups. Statistical significance was set at *P*<0.05 and indicated in the Figures reporting the data analysis. Beanplots, Mosaicplots and Forestplots were generated using R ver. 3.1.3 (software freely available at the website https://www.r-project.org/). The meta-analysis was conducted using R and the package metafor ver. 1.9–5 (freely available at the website http://www.metafor-project.org/doku.php) adopting the method of restricted maximum-likelihood estimator and a random-effect meta-regression model.

## Abbreviations

LHON: Leber's hereditary optic neuropathy
CSC: cigarette smoke condensate
ROS: reactive oxygen species
mtDNA: mitochondrial DNA
CS: citrate synthase
SDHA: subunit A of succinate dehydrogenase
NRF-1: nuclear respiratory factor-1
OXPHOS: oxidative phosphorylation
AhR: aryl hydrocarbon receptor
Nrf2: Nuclear factor erythroid 2-related factor 2
MnSOD: manganese superoxide dismutase
NQO1: NADPH dehydrogenase quinone 1
Prx III: peroxiredoxin III
qRT-PCR: Quantitative Real-Time PCR
OCR: Oxygen consumption rate

## References

[bib1] 1Yu-Wai-Man P, Griffiths PG, Brown DT, Howell N, Turnbull DM, Chinnery PF. The epidemiology of Leber hereditary optic neuropathy in the North East of England. Am J Hum Genet 2003; 72: 333–339. 12518276 10.1086/346066 PMC379226

[bib2] 2Mascialino B, Leinonen M, Meier T. Meta-analysis of the prevalence of Leber hereditary optic neuropathy mtDNA mutations in Europe. Eur J Ophthalmol 2012; 22: 461–465. 21928272 10.5301/ejo.5000055

[bib3] 3Carelli V, Ross-Cisneros FN, Sadun AA. Mitochondrial dysfunction as a cause of optic neuropathies. Prog Retin Eye Res 2004; 23: 53–89. 14766317 10.1016/j.preteyeres.2003.10.003

[bib4] 4Yu-Wai-Man P, Griffiths PG, Chinnery PF. Mitochondrial optic neuropathies - disease mechanisms and therapeutic strategies. Prog Retin Eye Res 2011; 30: 81–114. 21112411 10.1016/j.preteyeres.2010.11.002 PMC3081075

[bib5] 5Carelli V, La Morgia C, Valentino ML, Barboni P, Ross-Cisneros FN, Sadun AA. Retinal ganglion cell neurodegeneration in mitochondrial inherited disorders. Biochim Biophys Acta 2009; 1787: 518–528. 19268652 10.1016/j.bbabio.2009.02.024

[bib6] 6Maresca A, la Morgia C, Caporali L, Valentino ML, Carelli V. The optic nerve: a "mito-window" on mitochondrial neurodegeneration. Mol Cell Neurosci 2013; 55: 62–76. 22960139 10.1016/j.mcn.2012.08.004 PMC3629569

[bib7] 7Sadun AA, La Morgia C, Carelli V. Mitochondrial optic neuropathies: our travels from bench to bedside and back again. Clin Experiment Ophthalmol 2013; 41: 702–712. 23433229 10.1111/ceo.12086

[bib8] 8Pan BX, Ross-Cisneros FN, Carelli V, Rue KS, Salomao SR, Moraes-Filho MN et al. Mathematically modeling the involvement of axons in Leber's hereditary optic neuropathy. Invest Ophthalmol Vis Sci 2012; 53: 7608–7617. 23060142 10.1167/iovs.12-10452 PMC3495603

[bib9] 9Barboni P, Carbonelli M, Savini G, Ramos Cdo V, Carta A, Berezovsky A et al. Natural history of Leber's hereditary optic neuropathy: longitudinal analysis of the retinal nerve fiber layer by optical coherence tomography. Ophthalmology 2010; 117: 623–627. 20031228 10.1016/j.ophtha.2009.07.026

[bib10] 10Barboni P, Savini G, Valentino ML, La Morgia C, Bellusci C, De Negri AM et al. Leber's hereditary optic neuropathy with childhood onset. Invest Ophthalmol Vis Sci 2006; 47: 5303–5309. 17122117 10.1167/iovs.06-0520

[bib11] 11Yu-Wai-Man P, Bateman DE, Hudson G, Griffiths PG, Chinnery PF. Leber hereditary optic neuropathy presenting in a 75-year-old man. J Neuroophthalmol 2008; 28: 155. 18562849 10.1097/WNO.0b013e3181772db4

[bib12] 12Carelli V, La Morgia C, Valentino ML, Rizzo G, Carbonelli M, De Negri AM et al. Idebenone treatment in Leber's hereditary optic neuropathy. Brain 2011; 134: e188. 21810891 10.1093/brain/awr180

[bib13] 13Carelli V, Giordano C, d'Amati G. Pathogenic expression of homoplasmic mtDNA mutations needs a complex nuclear-mitochondrial interaction. Trends Genet 2003; 19: 257–262. 12711217 10.1016/S0168-9525(03)00072-6

[bib14] 14Carelli V, Achilli A, Valentino ML, Rengo C, Semino O, Pala M et al. Haplogroup effects and recombination of mitochondrial DNA: novel clues from the analysis of Leber hereditary optic neuropathy pedigrees. Am J Hum Genet 2006; 78: 564–574. 16532388 10.1086/501236 PMC1424694

[bib15] 15Hudson G, Carelli V, Spruijt L, Gerards M, Mowbray C, Achilli A et al. Clinical expression of Leber hereditary optic neuropathy is affected by the mitochondrial DNA-haplogroup background. Am J Hum Genet 2007; 81: 228–233. 17668373 10.1086/519394 PMC1950812

[bib16] 16Maresca A, Caporali L, Strobbe D, Zanna C, Malavolta D, La Morgia C et al. Genetic basis of mitochondrial optic neuropathies. Curr Mol Med 2014; (e-pub ahead of print 10 October 2014). 10.2174/1566524014666141010132627 25323873

[bib17] 17Giordano C, Montopoli M, Perli E, Orlandi M, Fantin M, Ross-Cisneros FN et al. Oestrogens ameliorate mitochondrial dysfunction in Leber's hereditary optic neuropathy. Brain 2011; 134: 220–234. 20943885 10.1093/brain/awq276 PMC3025718

[bib18] 18Giordano C, Iommarini L, Giordano L, Maresca A, Pisano A, Valentino ML et al. Efficient mitochondrial biogenesis drives incomplete penetrance in Leber's hereditary optic neuropathy. Brain 2014; 137: 335–353. 24369379 10.1093/brain/awt343 PMC3914475

[bib19] 19Arnold S, Victor MB, Beyer C. Estrogen and the regulation of mitochondrial structure and function in the brain. J Steroid Biochem Mol Biol 2012; 131: 2–9. 22326731 10.1016/j.jsbmb.2012.01.012

[bib20] 20Bianco A, Martínez-Romero I, Bisceglia L, D'Agruma L, Favia P, Ruiz-Pesini E et al. Mitochondrial DNA copy number differentiates the Leber's hereditary optic neuropathy affected individuals from the unaffected mutation carriers. Brain 2015 (in press). 10.1093/brain/awv216 26209315

[bib21] 21Istikharah R, Tun AW, Kaewsutthi S, Aryal P, Kunhapan B, Katanyoo W et al. Identification of the variants in PARL, the nuclear modifier gene, responsible for the expression of LHON patients in Thailand. Exp Eye Res 2013; 116: 55–57. 23973714 10.1016/j.exer.2013.08.007

[bib22] 22Hudson G, Keers S, Yu Wai Man P, Griffiths P, Huoponen K, Savontaus ML et al. Identification of an X-chromosomal locus and haplotype modulating the phenotype of a mitochondrial DNA disorder. Am J Hum Genet 2005; 77: 1086–1091. 16380918 10.1086/498176 PMC1285165

[bib23] 23Shankar SP, Fingert JH, Carelli V, Valentino ML, King TM, Daiger SP et al. Evidence for a novel x-linked modifier locus for leber hereditary optic neuropathy. Ophthalmic Genet 2008; 29: 17–24. 18363168 10.1080/13816810701867607

[bib24] 24Ji Y, Jia X, Li S, Xiao X, Guo X, Zhang Q. Evaluation of the X-linked modifier loci for Leber hereditary optic neuropathy with the G11778A mutation in Chinese. Mol Vis 2010; 16: 416–424. 20300564 PMC2838738

[bib25] 25Kirkman MA, Yu-Wai-Man P, Korsten A, Leonhardt M, Dimitriadis K, De Coo IF et al. Gene-environment interactions in Leber hereditary optic neuropathy. Brain 2009; 132: 2317–2326. 19525327 10.1093/brain/awp158 PMC2732267

[bib26] 26Ghelli A, Porcelli AM, Zanna C, Vidoni S, Mattioli S, Barbieri A et al. The background of mitochondrial DNA haplogroup J increases the sensitivity of Leber's hereditary optic neuropathy cells to 2,5-hexanedione toxicity. PLoS One 2009; 4: e7922. 19936068 10.1371/journal.pone.0007922 PMC2774515

[bib27] 27Sadun AA, Carelli V, Salomao SR, Berezovsky A, Quiros PA, Sadun F et al. Extensive investigation of a large Brazilian pedigree of 11778/haplogroup J Leber hereditary optic neuropathy. Am J Ophthalmol 2003; 136: 231–238. 12888043 10.1016/s0002-9394(03)00099-0

[bib28] 28Talhout R, Schulz T, Florek E, van Benthem J, Wester P, Opperhuizen A. Hazardous compounds in tobacco smoke. Int J Environ Res Public Health 2011; 8: 613–628. 21556207 10.3390/ijerph8020613 PMC3084482

[bib29] 29Beretta S, Mattavelli L, Sala G, Tremolizzo L, Schapira AH, Martinuzzi A et al. Leber hereditary optic neuropathy mtDNA mutations disrupt glutamate transport in cybrid cell lines. Brain 2004; 127: 2183–2192. 15342361 10.1093/brain/awh258

[bib30] 30Floreani M, Napoli E, Martinuzzi A, Pantano G, De Riva V, Trevisan R et al. Antioxidant defences in cybrids harboring mtDNA mutations associated with Leber's hereditary optic neuropathy. FEBS J 2005; 272: 1124–1135. 15720387 10.1111/j.1742-4658.2004.04542.x

[bib31] 31Rico de Souza A, Zago M, Pollock SJ, Sime PJ, Phipps RP, Baglole CJ. Genetic ablation of the aryl hydrocarbon receptor causes cigarette smoke-induced mitochondrial dysfunction and apoptosis. J Biol Chem 2011; 286: 43214–43228. 21984831 10.1074/jbc.M111.258764 PMC3234839

[bib32] 32Taguchi K, Motohashi H, Yamamoto M. Molecular mechanisms of the Keap1–Nrf2 pathway in stress response and cancer evolution. Genes Cells 2011; 16: 123–140. 21251164 10.1111/j.1365-2443.2010.01473.x

[bib33] 33Smith PR, Cooper JM, Govan GG, Harding AE, Schapira AH. Smoking and mitochondrial function: a model for environmental toxins. Q J Med 1993; 86: 657–660. 8255963 10.1093/qjmed/86.10.657

[bib34] 34van der Toorn M, Slebos DJ, de Bruin HG, Leuvenink HG, Bakker SJ, Gans RO et al. Cigarette smoke-induced blockade of the mitochondrial respiratory chain switches lung epithelial cell apoptosis into necrosis. Am J Physiol Lung Cell Mol Physiol 2007; 292: L1211–L1218. 17209140 10.1152/ajplung.00291.2006

[bib35] 35Jia L, Liu Z, Sun L, Miller SS, Ames BN, Cotman CW et al. Acrolein, a toxicant in cigarette smoke, causes oxidative damage and mitochondrial dysfunction in RPE cells: protection by (R)-alpha-lipoic acid. Invest Ophthalmol Vis Sci 2007; 48: 339–348. 17197552 10.1167/iovs.06-0248 PMC2597695

[bib36] 36Salem AF, Al-Zoubi MS, Whitaker-Menezes D, Martinez-Outschoorn UE, Lamb R, Hulit J et al. Cigarette smoke metabolically promotes cancer, via autophagy and premature aging in the host stromal microenvironment. Cell Cycle 2013; 12: 818–825. 23388463 10.4161/cc.23722 PMC3610729

[bib37] 37Pryor WA, Arbour NC, Upham B, Church DF. The inhibitory effect of extracts of cigarette tar on electron transport of mitochondria and submitochondrial particles. Free Radic Biol Med 1992; 12: 365–372. 1317324 10.1016/0891-5849(92)90085-u

[bib38] 38Raza H, John A, Nemmar A. Short-term effects of nose-only cigarette smoke exposure on glutathione redox homeostasis, cytochrome P450 1A1/2 and respiratory enzyme activities in mice tissues. Cell Physiol Biochem 2013; 31: 683–692. 23711494 10.1159/000350087

[bib39] 39Lee HM, Hallberg LM, Greeley GH Jr, Englander EW. Differential inhibition of mitochondrial respiratory complexes by inhalation of combustion smoke and carbon monoxide, *in vivo*, in the rat brain. Inhal Toxicol 2010; 22: 770–777. 20429857 10.3109/08958371003770315 PMC3398809

[bib40] 40DiMauro S, Schon EA. Mitochondrial respiratory-chain diseases. N Engl J Med 2003; 348: 2656–2668. 12826641 10.1056/NEJMra022567

[bib41] 41Rossignol R, Faustin B, Rocher C, Malgat M, Mazat JP, Letellier T. Mitochondrial threshold effects. Biochem J 2003; 370: 751–762. 12467494 10.1042/BJ20021594 PMC1223225

[bib42] 42Jakoby WB, Ziegler DM. The enzymes of detoxication. J Biol Chem 1990; 265: 20715–20718. 2249981

[bib43] 43Anzenbacher P, Anzenbacherová E. Cytochromes P450 and metabolism of xenobiotics. Cell Mol Life Sci 2001; 58: 737–747. 11437235 10.1007/PL00000897 PMC11337355

[bib44] 44Sheeran FL, Rydström J, Shakhparonov MI, Pestov NB, Pepe S. Diminished NADPH transhydrogenase activity and mitochondrial redox regulation in human failing myocardium. Biochim Biophys Acta 2010; 1797: 1138–1148. 20388492 10.1016/j.bbabio.2010.04.002

[bib45] 45King MP, Attardi G. Human cells lacking mtDNA: repopulation with exogenous mitochondria by complementation. Science 1989; 246: 500–503. 2814477 10.1126/science.2814477

[bib46] 46Kwon J, Han E, Bui CB, Shin W, Lee J, Lee S et al. Assurance of mitochondrial integrity and mammalian longevity by the p62-Keap1-Nrf2-Nqo1 cascade. EMBO Rep 2012; 13: 150–156. 22222206 10.1038/embor.2011.246 PMC3271336

[bib47] 47Piantadosi CA, Carraway MS, Babiker A, Suliman HB. Heme oxygenase-1 regulates cardiac mitochondrial biogenesis via Nrf2-mediated transcriptional control of nuclear respiratory factor-1. Circ Res 2008; 103: 1232–1240. 18845810 10.1161/01.RES.0000338597.71702.ad PMC2694963

[bib48] 48Cantó C, Gerhart-Hines Z, Feige JN, Lagouge M, Noriega L, Milne JC et al. AMPK regulates energy expenditure by modulating NAD+ metabolism and SIRT1 activity. Nature 2009; 458: 1056–1060. 19262508 10.1038/nature07813 PMC3616311

[bib49] 49Cerutti R, Pirinen E, Lamperti C, Marchet S, Sauve AA, Li W et al. NAD(+)-dependent activation of Sirt1 corrects the phenotype in a mouse model of mitochondrial disease. Cell Metab 2014; 19: 1042–1049. 24814483 10.1016/j.cmet.2014.04.001 PMC4051987

[bib50] 50Khan NA, Auranen M, Paetau I, Pirinen E, Euro L, Forsström S et al. Effective treatment of mitochondrial myopathy by nicotinamide riboside, a vitamin B3. EMBO Mol Med 2014; 6: 721–731. 24711540 10.1002/emmm.201403943 PMC4203351

[bib51] 51Carelli V, D'Adamo P, Valentino ML, La Morgia C, Ross-Cisneros F, Caporali L et al. Parsing the differences in affected with LHON: genetic versus environmental triggers of disease conversion. Brain 2015 (in press). 10.1093/brain/awv339 PMC6080496 26657166

[bib52] 52Pfaffl MW. A new mathematical model for relative quantification in real-time RT-PCR. Nucleic Acids Res 2001; 29: e45. 11328886 10.1093/nar/29.9.e45 PMC55695

