# Supplementary material for: Cigarette toxicity triggers Leber's hereditary optic neuropathy by affecting mtDNA copy number, oxidative phosphorylation and ROS detoxification pathways
Source: Cell Death Dis. 2015 Dec 17;6(12):e2021–. doi: 10.1038/cddis.2015.364 (PMC4720897; doi:10.1038/cddis.2015.364)
Supplement: Supplementary Materials and Methods [file cddis2015364x1.docx]

**Supplementary Materials and Methods**

**Cell viability**

Cell viability was assessed evaluating the ability of protein dye sulforhodamine B (SRB) to bind cellular proteins^1^. In brief, 1.2x10^4^ cells were seed in each well of a Multiwell FALCON Becton Dickinson plate in the presence of medium (DMEM containing 25 mM glucose). The medium was removed after 24 hours and each well was washed with PBS 1%. Then fibroblasts were incubated with different concentration of CSC in DMEM-Glucose. After 72 hrs, cell monolayer were fixed with 50% trichloroacetic acid (Sigma Aldrich) and stained for 30 min with SRB 0.4%. The excess dye was removed by washing repeatedly with 1% (vol/vol) acetic acid and the protein-bound dye was dissolved in 10 mM Tris-base solution for OD determination at 570 nm using a microplate reader (VersaMax Microplate Reader Molecular Devices). Cell viability was calculated by relating the value observed in the CSC treated cells to that of untreated samples (100%).

**Assessment of mitochondrial bioenergetics**

The rate of mitochondrial ATP synthesis was measured in digitonin-permeabilized fibroblasts by using the luciferin/luciferase assay, according to a previously published method^2^, with minor modifications^3^. Briefly, after trypsinization, cells (10x10^6^/ml) were suspended in a buffer containing 150 mM KCl, 25 mM Tris-HCl, 2 mM EDTA, 0.1% bovine serum albumin, 10 mM potassium phosphate, 0.1 mM MgCl_2_, pH 7.4, kept at room temperature for 15 min, then incubated with 50 μg/ml digitonin until 90-100% of cells were positive to Trypan blue staining. Aliquots of 3x10^5^ permeabilized cells were incubated in the same buffer in presence of the adenylate kinase inhibitor P1,P5-di(adenosine-5') pentaphosphate (0.1 mM) and the Complex I substrates (1 mM malate plus 1 mM pyruvate). After addition of 0.1 mM ADP, chemiluminescence was determined as a function of time with a luminometer. The chemiluminescence signal was calibrated with an internal ATP standard after addition of 10 μM oligomycin. The titration of CSC was performed adding CSC directly in cuvette before starting the ATP synthesis reaction. The rates of ATP synthesis were normalized to protein contents^4^ and expressed as percentage of activity respect the non-treated samples. Each value was the mean based on at least 6 determinations from four different cell lines.

Oxygen consumption rate (OCR) in adherent cells was measured with an XF24 Extracellular Flux Analyzer (Seahorse Bioscience, Billerica, MA, USA), as previously described^3^ with minor variations. Brieﬂy, fibroblasts were seeded in XF24 cell culture microplates (Seahorse Bioscience) at 3x10^4^ cells/well in 250 µl of medium and incubated at 37 °C in 5% CO_2_ for 24 hrs. The day after, the assays were initiated following the manufacturer’s instructions by replacing the growth medium in each well with 525 μl of un-buffered DMEM- glucose pre-warmed at 37 °C. Cells were incubated at 37 °C for 60 min to allow temperature and pH equilibration. After an OCR baseline measurement, 75 µl of medium containing different amount of CSC (0, 24, 48, 72 µg/ml) were added and oxygen consumption was measured. Subsequently, the minimum oxygen consumption was determined adding 1 µM oligomycin and the maximal respiration rate was assessed adding 1 µM FCCP. At the end of the experiment the non-mitochondrial oxygen consumption was evaluated adding both 1 µM rotenone and antimycin. Protein content for each well was determined using the colorimetric sulforhodamine B assay, as previously detailed^1^. Data are expressed as percentage of residual maximal respiration capacity respect to untreated cells.

Respiratory complex activity was measured on isolated mitochondria from fibroblasts treated for 72 hrs with 80 µg/ml of CSC. Fibroblasts grown to confluency in four-six 150 cm^2^ tissue culture flasks, were suspended in 1.5 ml of 250 mM sucrose, 2 mM Hepes, 0.1 mM EGTA, pH 7.4 and homogenized by 20 passes in a homogenizer with a tight-fitting power driven Teflon plunger. The homogenate was centrifuged for 10 min at 600 x g at 4°C and the mitochondria-enriched supernatant was collected and centrifuged for 10 min at 11.000 x g at 4°C. The pellet was washed in 1 ml of 25 mM potassium phosphate pH 7.2, 5 mM MgCl_2_, and centrifuged for 10 min at 11.000 x g at 4°C. The mitochondrial pellet was suspended in 25 mM potassium phosphate, 5 mM MgCl_2_, pH7.2. For Complex I activity measurement mitochondria were subjected to three cycles of freezing and thawing, and suspended in a buffer containing 25 mM potassium phosphate pH 7.2, 5 mM MgCl_2_, 2 mM KCN, 2.5 mg/ml bovine serum albumin, 2 mg/ml antimycin A, 65 mM ubiquinone1, 130 mM NADH at 30 °C (ref. 5). Activity was measured for 3-4 min before addition of 2 mg/ml rotenone following the decrease in absorbance, due to the oxidation of NADH, at 340-425 nm. The rotenone insensitive rate of NADH oxidation was measured and subtracted. The Complex IV (cytochrome c oxidase) activity was measured by following the oxidation of cytochrome c at 550-540 nm. The mitochondrial fraction was added to reaction buffer containing 20 mM potassium phosphate (pH 7.0), 0.45 mM *n*-dodecyl-beta-D-maltoside and 15 mM cytochrome c (reduced) at 30 °C. Both activities were normalized to the mitochondrial proteins used in the assay.

Total ATP level was determined in fibroblasts treated for 72 hrs with different amounts of CSC, by using the luciferin/luciferase assay in GloMax 20/20 Luminometer (Promega) as described elsewhere^2,6^.

L-lactate production in the culture medium was measured spectrophotometrically monitoring the formation of NADH following its oxidation to pyruvate in the presence of lactate dehydrogenase (LDH) (ref. 7). Briefly, aliquots of the supernatant were added to a reaction mixture containing 1 mM NAD^+^, 20 U heart bovine LDH (Pierce), 0.2 M glycine and 0.15 M hydrazine, pH 9.5. Reaction was carried out at 25°C and followed for approximately 15 min.

**SDS-PAGE and Western blotting**

Total proteins were extracted from 1-2 x 10^6^ fibroblasts by homogenization in 220 mM mannitol, 70 mM sucrose, 20 mM Tris, 1mM EDTA, 5 mM EGTA, 5 mM MgCl_2_, pH 7.4.

Equal quantities of proteins (10 μg per lane) were separated by electrophoresis in a 4-12% SDS-polyacrylamide gradient gel (Criterion TM XT Precasted Gels Biorad) and electrotransferred onto a PVDF membrane (Millipore). The membrane was washed for 30 min in PBS 1X, 0.1% Tween 20, blocked for 1 hrs with 5% (w/v) nonfat dry milk in PBS 1X, 0.1% Tween 20 and then incubated overnight at 4°C in 1% (w/v) nonfat dry milk in PBS 1X, 0.1% Tween 20, with different rabbit antibodies. They were: anti-human citrate synthase (CS, 1:40000, Sigma Aldrich); anti-human succinate dehydrogenase subunit A (SDHA, 1:1000, Cell Signaling); anti-human Nuclear Respiratory Factor 1 (NRF-1, 1:8000, Santa Cruz); anti-human Manganese superoxide dismutase (MnSOD, 1:250000, Stressgene); anti-human Peroxiredoxin III (PrxIII, 1:150000, Ab Frontier-Seoul Korea); anti-human Nuclear factor erythroid 2-related factor 2, (Nrf2, 1:800, Millipore); anti-human NAD(P)H quinone oxidoreductase 1 (NQO1, 1:800, Calbiochem); anti-human Arylhydrocarbon receptor (AhR, 1:1000 Millipore), anti-human glutathione-S-transferase mu 5 (GST-M5, 1:10000, Thermo Scientific), anti-human glutathione reductase (GR, 1:50000, Pierce), anti-human β-actin (1:40000, Sigma Aldrich). Membranes were washed three times (10 min each one) with PBS 1X, 0.1% Tween 20 and three times (10 min each one) with 5% (w/vol) milk powder in PBS 1X, 0.1% Tween 20. Then they were incubated at room temperature for one hour, with secondary goat anti-rabbit (Santa Cruz), antibodies. They were a used at dilution 1:5000 for SDHA, Nrf2 AhR and NQO1; 1:10000 for CS, MnSOD, PrxIII, GST-M5, GR, and β-act; 1:15000 for NRF-1. Then followed three washes (10 min each one) with 5% (w/v) milk powder in PBS 1X, 0.1% Tween 20 and three washes (10 min each one) with PBS 1X, 0.1% Tween 20. The immunoreactivity was detected using the chemiluminescent substrate ECL (Amersham Biosciences). Quantitative analyses of the blots were performed using the Quantity One software (BioRad) in the Chemi Doc System (BioRad). The densitometric value of OD units of each band was then related to the OD units of the corresponding band of the β-actin. Preliminary experiments were performed to ensure that the observed values for each band were in the range of linearity.

**Quantification of carbonylated proteins**

Carbonylated proteins were quantified by means of the OxiBlot Protein Oxidation Detection Kit (Merck-Millipore), following the manufacturer's instruction. Briefly, equal quantities of proteins (10 μg) were treated with 460 mM β-Mercaptoetanol and 2.4% SDS in a final volume of 12.5 μl. The carbonyl groups in the protein side chains were derivatizated incubating for 20 min at 30 °C with 2,4-dinitrophenylhydrazine (DNPH) producing 2,4-dinitrophenylhydrazone. (DNP hydrazone) After blocking the derivatizated reaction with neutralization solution for 5 min, protein samples were run on a 4-12% SDS-polyacrylamide gradient gel (Criterion TM XT Precasted Gels Biorad) followed by western blotting. It was performed as described above except that instead of milk, the blocking agent was 3% BSA in PBS 1X, 0.1% Tween 20. The membrane was treated overnight with a specific antibody against DNP-hydrazone (diluted 1:400 with 1% BSA in 1X PBS, 0.1% Tween 20) and for one hour with the secondary antibody (diluted 1:1000 in 3% BSA in 1X PBS, 0.1% Tween 20). Afterwards the membrane was washed two times (20 minute each one) at 60 °C in 100 mM β-Mercaptoetanol, 2% SDS, 62.5mM Tris-HCl, pH 6.7 to strip primary antibody, and reprobed for β-actin (rabbit, 1:20000, Sigma Aldrich) used as reference protein. Detection of immunoreactive bands and quantitative analysis were performed as described in western blot paragraph.

**Supplementary References**

1. Vichai V, Kirtikara K. Sulforhodamine B colorimetric assay for cytotoxicity screening. Nat Protoc 2006; 1:1112-6.

2, Manfredi G, Yang L, Gajewski CD, Mattiazzi M. Measurements of ATP in mammalian cells. Methods 2002; 26:317-26.

3. Giorgio V, Petronilli V, Ghelli A, Carelli V, Rugolo M, Lenaz G, Bernardi P. The effects of idebenone on mitochondrial bioenergetics Biochim Biophys Acta 2012; 1817:363-9.

4. Bradford MM. A rapid and sensitive method for the quantitation of microgram quantities of protein utilizing the principle of protein-dye binding. Anal Biochem. 1976; 72: 248-54.

5. Kirby DM, Thorburn DR, Turnbull DM, Taylor RW. Biochemical assays of respiratory chain complex activity. Methods Cell Biol. 2007; 80:93-119.

6. Zanna C, Ghelli A, Porcelli AM, Martinuzzi A, Carelli V, Rugolo M. Caspase-independent death of Leber's hereditary optic neuropathy cybrids is driven by energetic failure and mediated by AIF and Endonuclease G. Apoptosis 2005; 10:997-1007.

7. Walz W, Mukerji S. Lactate production and release in cultured astrocytes. Neurosci Lett 1988; 86:296-300.
