# Supplementary material for: Cigarette toxicity triggers Leber's hereditary optic neuropathy by affecting mtDNA copy number, oxidative phosphorylation and ROS detoxification pathways
Source: Cell Death Dis. 2015 Dec 17;6(12):e2021–. doi: 10.1038/cddis.2015.364 (PMC4720897; doi:10.1038/cddis.2015.364)
Supplement: Supplementary Figure Legends [file cddis2015364x2.docx]

**Supplementary Figure Legends**

**Supplementary Figure 1. Forest plots of the metanalysis. A)** Differences in the distribution of smokers between affected individuals and unaffected mutation carriers in the two cohorts. **B)** Differences in mtDNA copy number in both the smokers and non-smokers in the affected groups.

**Supplementary Figure 2. Effect of CSC on the viability of fibroblasts from LHON affected patients, unaffected mutation carriers and controls**. Cell viability was assessed with the SRB cytotoxic assay. Results are the average of measures performed on 5 samples for each cell type. Histogram reports the mean value (± SEM) normalized with respect to that of the untreated sample (UN). * p (treated vs untreated)<0.05. In all cell types CSC did not affect cell viability up to 120 μg/ml. 1% DMSO (CSC solvent) did not affect cell viability.
