# Supplementary figures and images for: Cigarette toxicity triggers Leber's hereditary optic neuropathy by affecting mtDNA copy number, oxidative phosphorylation and ROS detoxification pathways
Source: Cell Death Dis. 2015 Dec 17;6(12):e2021–. doi: 10.1038/cddis.2015.364 (PMC4720897; doi:10.1038/cddis.2015.364)

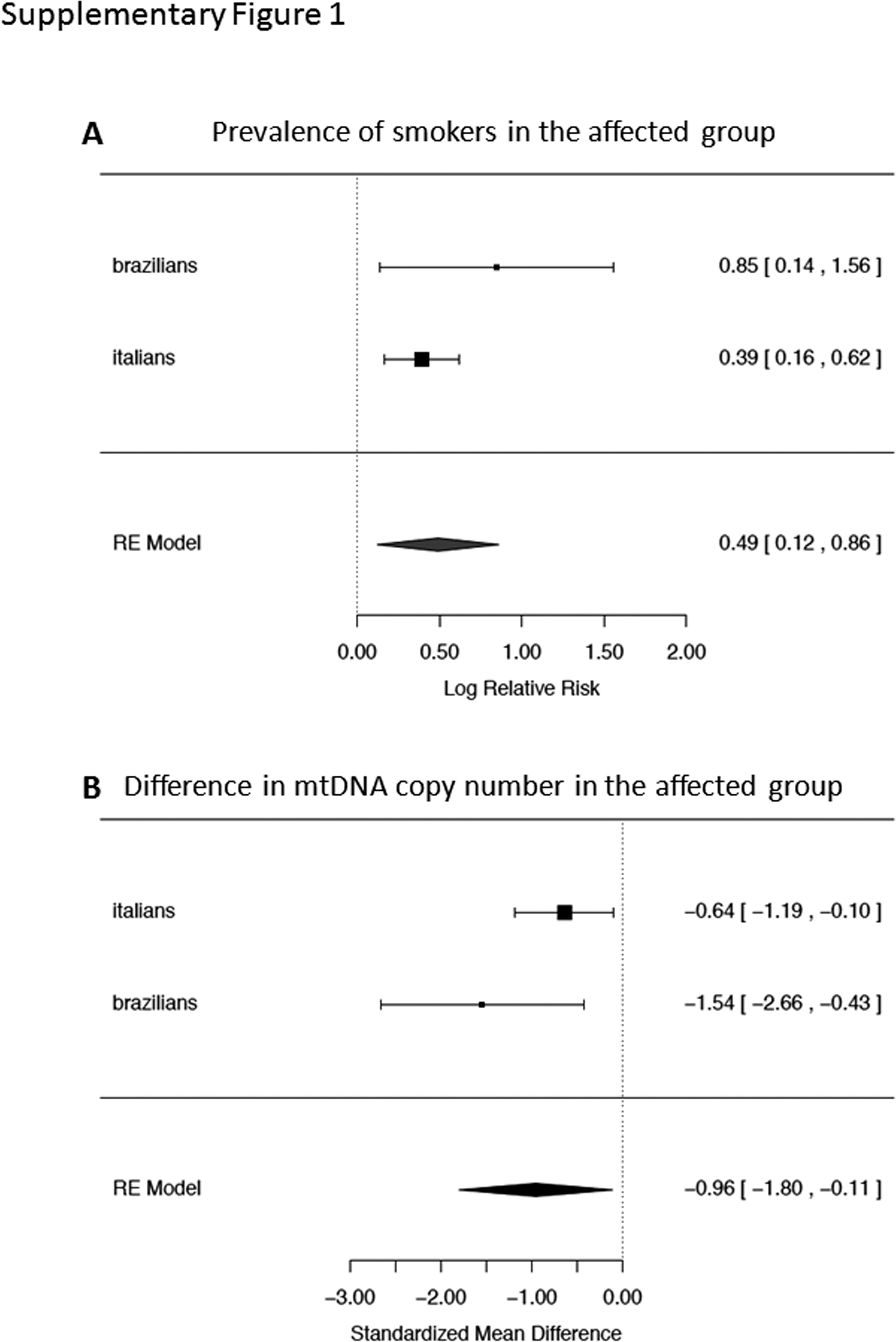

Supplement: Supplementary Figure 1 [file cddis2015364x3.tif]

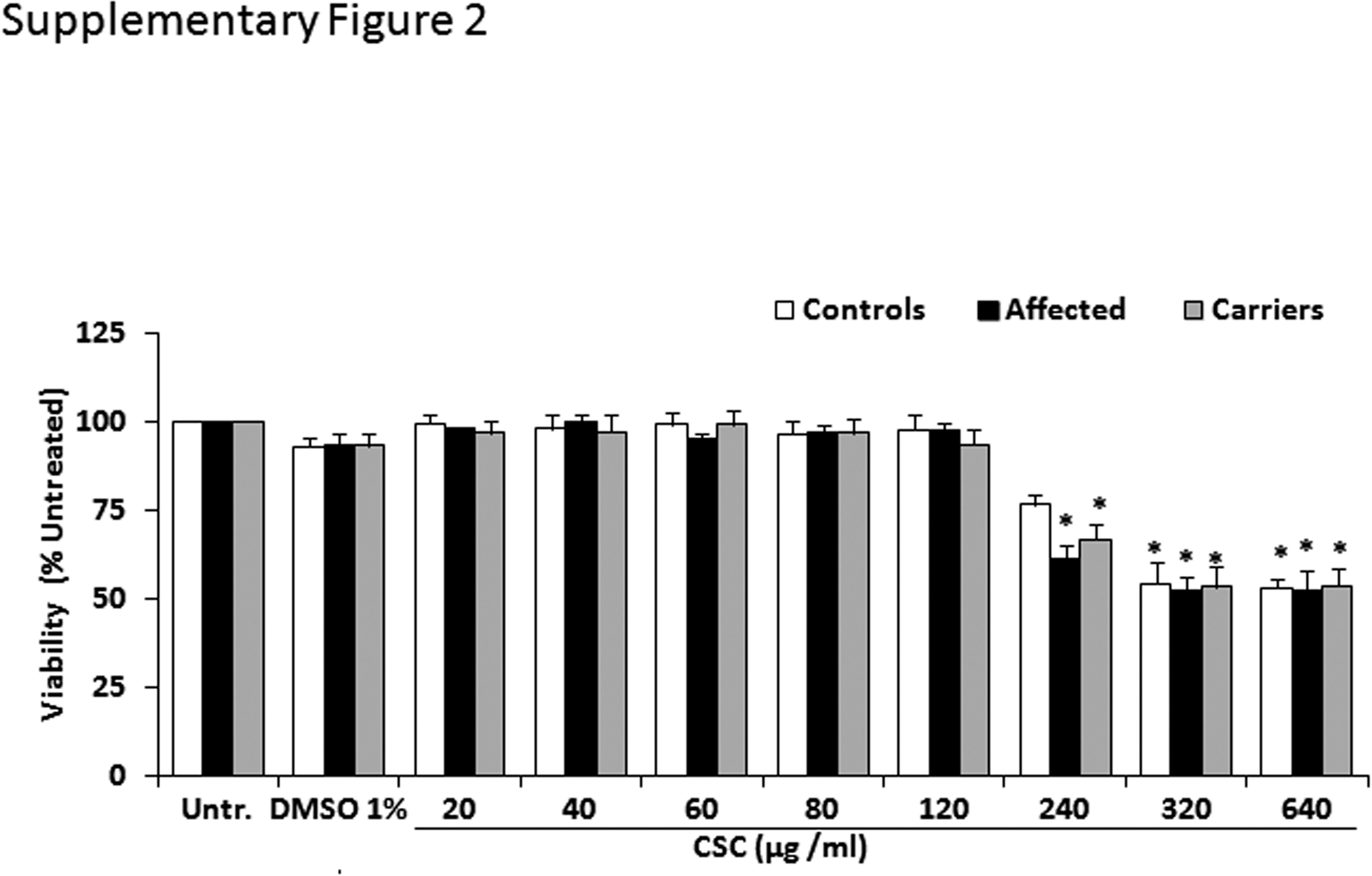

Supplement: Supplementary Figure 2 [file cddis2015364x4.tif]
